# Approaches to priority identification in digital health in ten countries of the Global Digital Health Partnership

**DOI:** 10.3389/fdgth.2022.968953

**Published:** 2022-09-16

**Authors:** Fidelia Cascini, Gerardo Altamura, Giovanna Failla, Andrea Gentili, Valeria Puleo, Andriy Melnyk, Francesco Andrea Causio, Walter Ricciardi

**Affiliations:** Department of Life Sciences and Public Health, Università Cattolica del Sacro Cuore, Roma, Italy

**Keywords:** digital health, GDHP, priorities identification, interoperability, cross-countries

## Abstract

**Background:**

To promote shared digital health best practices in a global context, as agreed within the Global Digital Health Partnership (GDHP), one of the most important topics to evaluate is the ability to detect what participating countries believe to be priorities suitable to improve their healthcare systems. No previously published scientific papers investigated these aspects as a cross-country comparison.

**Objective:**

The aim of this paper is to present results concerning the priorities identification section of the Evidence and Evaluation survey addressed to GDHP members in 2021, comparing countries’ initiatives and perspectives for the future of digital health based on internationally agreed developments.

**Methods:**

This survey followed a cross-sectional study approach. An online survey was addressed to the stakeholders of 29 major countries.

**Results:**

Ten out of 29 countries answered the survey. The mean global score of 3.54 out of 5, calculated on the whole data set, demonstrates how the global attention to a digital evolution in health is shared by most of the evaluated countries.

**Conclusion:**

The resulting insights on the differences between digital health priority identification among different GDHP countries serves as a starting point to coordinate further progress on digital health worldwide and foster evidence-based collaboration.

## Introduction

In 2019, the World Health Organization (WHO) started developing a framework for the adoption of digital innovations and technology in healthcare. The WHO recommendations on digital interventions in healthcare promotes assessment based on “benefits, harms, acceptability, feasibility, resource use and equity considerations,” and views these tools as still very much that—tools—in the journey to achieving universal health coverage and sustainability (1).

Following Mescó et al. definition, “Digital health” is considered as “the cultural transformation of how disruptive technologies that provide digital and objective data accessible to both caregivers and patients leads to an equal level doctor-patient relationship with shared decision-making and the democratization of care,” initiating changes in providing care and practicing medicine (2). As technological innovations become inseparable from healthcare and as healthcare systems worldwide are becoming financially unsustainable, a paradigm shift might be considered imminent.

To investigate this process, the global summit for digital health named Global Digital Health Partnership (GDHP) was internationally launched in 2018 (3), pushed by the need of country governments to work to advance global digital health together. It is a collaboration of 33 countries and territories, the World Health Organization (WHO), Organisation for Economic Co-operation and Development (OECD), and The International Digital Health / AI Research Collaborative (I-DAIR), formed to support the effective implementation of digital health, exchange global best practices, and advance mutually beneficial projects. It is the world's only existing government-to-government global health Information Technology partnership, aiming to discover international best practices for the use of health data to advance health and health care, encourage networking and knowledge transfer, and facilitate horizon scanning to more accurately forecast emerging trends (4). The main evaluated topics of this partnership range from cyber security to interoperability, evidence and evaluation, policy environments, clinical and consumer engagement (5).

Within a few weeks of the Covid-19 outbreak, the following lockdowns accelerated the adoption of digital solutions at an unprecedented pace, creating unforeseen opportunities for scaling up alternative approaches to social and economic life (6). With a huge amount of critical situations to manage in an unpredictable context, the preparedness of countries has been crucial during the pandemic (7). The need for a fast response to be applied on the largest possible scale brought the public's attention back to digitalised healthcare. A daily experience of a digitalised prevention and control instrument can be found in digital Covid-19 certificates. Although they still have some issues that need to be addressed, it is a simple digital instrument that allows an increasing amount of people to cope with a viral pandemic in an easy and safe way (8).

The potential for data-driven technologies to revolutionise the delivery of healthcare has been much discussed over the past few years. However, delivering on this potential at scale across health systems is yet to be realised (9).

Even though every country has a different health care system, governance structure, and culture, all countries should use standard health data standards to allow, among other advantages, easier sharing of best practices, increased accessibility to healthcare services and safer assistance at an international level for foreign citizens. Countries and territories are at different stages of adoption but the implementation of these health data standards and their harmonization is crucial to promote the interoperability of health data across the globe (10).

In February 2019, the Evidence and Evaluation workstream of the Global Digital Health Partnership (GDHP) published one of their first papers. It provided an overview of the measurement frameworks and approaches to international benefits in addition to the benefits of digital health technologies and services among GDHP participant countries. In July 2020, a whitepaper was published on the development of standard benefits and outcome measurements (11) and was aimed at providing a common framework for the evaluation of digital health services and technologies among different countries that could be used for a comparison of results. All GDHP country participants were offered the opportunity to contribute to the Evidence and Evaluation workstream's whitepaper.

GDHP country experiences allowed the development of a Maturity Framework (12) to guide countries on how to proceed. The involved framework items are local and national data collection and use, data linkage, dataset access and released, open data. To reach maturity for the correct use of obtained health information, several conditions need to be in place to support maturity- such as Information Communication Technology (ICT) infrastructure and foundations in place, established governance models and public engagement.

Furthermore, the proposed questions aim to assess whether a pragmatic approach to improve health services through digital health has already been implemented and if the experience of other countries in the digitalization of healthcare could help in the process of identifying priorities.

Traditionally, the GDHP aims to explore the differences between countries in terms of digital health to 1 day reach a harmonization of developments through interoperability. Previous works (11, 13, 14) have left some questions unresolved and we are going to explore those through this study. In order to gather data and opinions from the wide range of the partnership stakeholders, GDHP can benefit of a typical qualitative tool, thus surveys addressed to its own members. This is one of the various opportunities of discussion that members can interact with. Surveys are widely used and participation of recipients is totally voluntary.

This paper focuses on the “priorities identification” section within the latest GDHP survey, thus derived from a wider and more inclusive document, yet to be published. To strategically develop digital health in a country, the identification of key priorities is the first approach for planning required actions (15). Priority setting is the process of making decisions about how best to allocate limited resources to improve population health (16). It is a complex and inherently political process that requires multiple stakeholders, decision-makers, and actors whose beliefs are often imperfectly aligned (17).

This paper addresses the results derived from the answers of 10 different member countries of the GDHP to our survey. Relative priorities for action within any country was determined by countries’ concerns and by balancing socio-economical, technological, political and educational interventions. It followed a top-down method by directly collecting representative bodies’ answers to the suggested topics (18, 19). The aim of this paper is to analyse the obtained results, highlighting how individual countries intend to address the digital shift of healthcare, reporting similarities and differences, encouraging the fundamental dialog required to follow the interoperability and sharing principles on which the GDHP project is based. As a consequence of GDHP expert group heterogeneity, our study enjoys a privileged point of view over the cooperation of countries with different socio-economic statuses for the digital transition of national health systems.

## Methods

### Rapid review of literature

To produce this survey, in order to methodologically measure the use and the acceptability of digital health interventions, a rapid review of literature was conducted during March and April 2021 to identify publications relating to the evaluation of a range of digital health technologies and innovations.

### Search strategy and data sources

Some of the included keywords in the search string were “digital health,” “health digitalisation,” “national plan,” “WHO framework,” “The digital competence framework,” “questionnaire,” “survey” and “acceptability” and they were related by Boolean operators.

Academic databases and search engines used included PubMed, Web of Science and Scopus. Grey literature research, using Google site search function, was conducted to identify related missing records. The questionnaire was then developed and provided to each GDHP country's representative.

The rapid review of the international literature concerned the evaluation methods and proximal measurements used in the field of digital health use and acceptability. Literature review data was supported by a nominal group consensus process to develop a pragmatic questionnaire to be administered to stakeholders in different countries who are considering performing digital health assessments. The full list of searched terms can be found in the “Supplementary Material” section.

### Inclusion and exclusion criteria

Only original papers written in English and with full texts available were included. Following the aim of this survey, only articles regarding the evaluation of use and acceptability of digital health services and technology and the digital transition of health, administering a questionnaire and published in a peer-reviewed scientific journal were included. In addition, a grey literature scoping review was performed to fill the resulting information gaps. The search was limited to literature published after 2010, with a focus on articles published after 2019's “Digital Competence Framework” release from the WHO.

### Study selection and data extraction

Articles were first assessed based on title and abstract then the full text was analysed by the researchers for all eligible articles. Data extraction was performed using a Microsoft Excel® for Windows spreadsheet. To standardize the data extraction process, the team prepared a predefined shared spreadsheet. Several qualitative and quantitative data were extracted from the original studies. Qualitative data recorded included the name of the first author, year, type of questionnaire, country and digital field. Quantitative data extracted included the number of recorded answers, readers’ compliance, evaluated fields and duration of the survey.

### Survey development

A structured multiple-choice questionnaire/survey was designed to gather data. The survey choices were extracted from research publications retrieved during the rapid review. In addition to answering the structured questions, respondents had the chance to elaborate their answers and to offer comments.

In this GDHP survey, government authorities are considered the main stakeholders. A list of involved responders can be found in Supplementary Figure S1.

GDHP participant countries, organisations and territories were invited to participate in the analysis by responding to the survey. There was only one response allowed per country or territory. The survey asked questions under six main sections: “Priorities identification,” “Relationship between national health plans and criteria for funds allocation,” “Evidence about the development of digital health services,” “Providing digital health evidence,” “Collecting Data” and “Strengthening and promoting digital health.” This paper focuses on the 1st section of the survey “priorities identification”.

The survey was submitted to 29 different worldwide distributed countries, ten of which forwarded their answers.

### Priorities identification

This survey section examines the current legislation on the matter and the existence of official documents about digital health priorities and specific plans to implement digital health technologies.

Relative priorities for action within any country was determined by countries concerns, by balancing socio-economical, technological, political, and educational interventions.

This section composed of 33 different questions (see Table 1) that were separated into 3 different main topics defined as follows:

• Topic 1: Health Technology Assessment (HTA) and Transformation Policies for the Digitalisation of Healthcare.
  o This investigated the importance given to the national HTA processes and eHealth strategies to enhance the digitalisation of healthcare services.
• Topic 2: Development of Digital Health Integration.
  o This examined the relevance attributed to the integration of different healthcare services and systems in addition to the architecture used for the interoperability of health data.
• Topic 3: Digitalisation of Healthcare Services
  o This investigated the attention respondents place on the implementation of a series of digital health services.

**Table 1:** Topics, identification codes, items.

Health Technology Assessment (HTA) and transformation policies for digitalisation healthcare	a1	National ehealth strategy
	a2	National ehealth system/platform
	a3	National digital health agency
	a4	Institutions/Organizations for digital health
	a5	National digital networking system
	a6	Cost-benefit assessment of digital technologies
	a7	Effectiveness of digital health innovation
Development of digital health integration	b1	Networks to exchange health records and documents
	b2	Integrated information system for prevention
	b3	Healthcare services network
	b4	Centralised network architecture
	b5	Decentralised architecture
	b6	Healthcare services network including pharmacies
	b7	Drug information system
Digitalisation of healthcare services	c1	Emergency support information systems
	c2	Ambulance monitoring systems using real-time dashboards
	c3	Development of digitalisation and increased use of technological infrastructures (smart hospitals)
	c4	Strengthening the digital monitoring system
	c5	Online booking for healthcare services
	c6	Online payments for healthcare services
	c7	Artificial intelligence for diagnostic healthcare
	c8	Artificial intelligence for population studies
	c9	Artificial intelligence for predictive models of prevention
	c10	Accessibility to health services
	c11	Delivery of healthcare
	c12	Telemedicine
	c13	Telenursing
	c14	Electronic patient portals
	c15	Electronic hospital discharge
	c16	Clinical decision- support system
	c17	Robotic use for care
	c18	Electronic patient reminder *via* mHealth
	c19	Digital health literacy

For each proposed item, respondents were requested to assign a value on a scale from 1 (very low) to 5 (very high) as the answer to the following question: “What are currently the digital health priority areas in your country?” A quantitative synthesis of the obtained answers was conducted after the answers were submitted.

### Statistics

A multivariate analysis was carried out using the biplot methodology to view how different key priorities behaved in different countries.

Multivariate statistical analyses were run with STATISTICA (StatSoft Inc.). The score dataset was then subjected to cluster analysis to assess which countries identified similar priorities. A discriminant analysis was run to define which countries best differentiated for the three examined topics. The confidence of discrimination was evaluated based on the *F*-test of significance of the partial regression coefficient of the last variable entering the model and using Wilks’ lambda. Mahalanobis distances were also calculated to compare the relative distances among topics. Canonical multiple discriminant analysis was used for graphical representation of the items’ distributions, by plotting the individual scores for the two principal functions obtained from the model. For each country, the respective contribution to the discrimination was examined using the standardised b coefficient.

The obtained findings derived from the results of this survey section will provide a view on national priorities regarding health digitalisation around the world.

## Results

Data elaborated in this section refer to a total of 33 proposed items, of which ten worldwide distributed countries were assigned a score ranging from 0 to 5.

**Figure 1:**
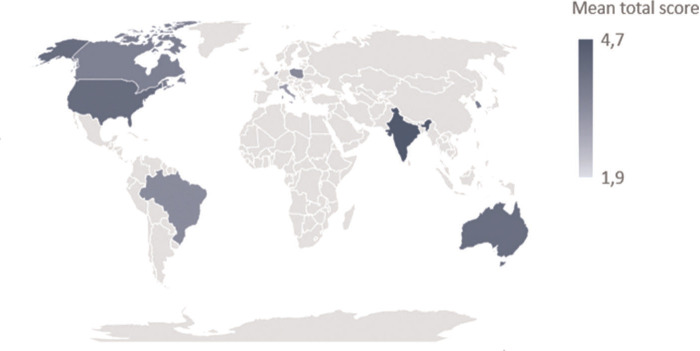
Cartogram representing the ten involved countries in this survey and the mean score attributed by them to the total of listed items.

Out of the twenty-nine countries that were reached out to, ten filled out the “priorities identification” section of the survey: three from Europe, three from Asia, two from North America, one from South America and one from Oceania (Figure 1). Seven questions concerned “Health Technology Assessment (HTA) and transformation policies for digitalisation of healthcare”, seven “Development of digital health integration”, nineteen “Digitalisation of healthcare services”.

As shown in Supplementary Table S1, all three topics had an intermediate score. Specifically, Topic 1 had a mean score of 3.64, followed by Topic 2 with a mean score of 3.58 and Topic 3 with a mean score of 3.49.

**Figure 2:**
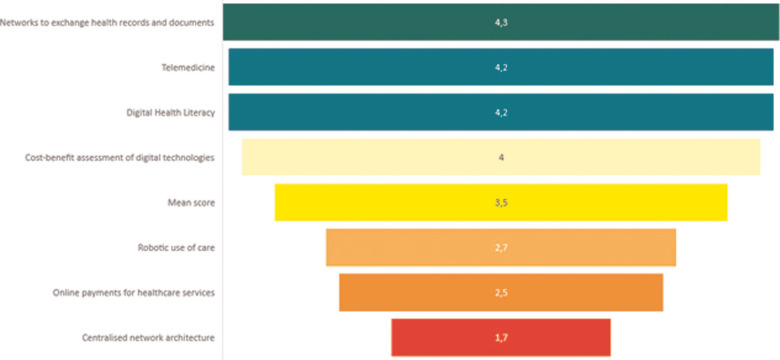
Most and least voted items (means) compared to mean total value.

After globally evaluating the survey, the field that obtained the highest score and thus the highest priority was the “development of digital health integration.” The most voted item within that field was “Networks to exchange health records and documents” (4.3). The second highest priority is the “digitalisation of healthcare services” with the items “Telemedicine” (4.2), “Digital health literacy” (4.2) and “Electronic hospital discharge” (4.1) as the most voted items. Finally, in the “HTA and transformation policies for digitalisation of healthcare” field, the most shared priority was the “cost-benefit assessment of digital technologies” (4.0) (Figure 2).

The least voted, and thus the lowest prioritized, item regarded the construction of a “centralised network architecture” (1.7) in the “Development of digital health integration” field. Most countries attributed higher votes to the “decentralised architecture” model, with a mean score of 3.9. Low priority was also attributed to “online payments for healthcare services” as seen by it having a mean score of 2.5 and to “robotic use for care,” with a mean score of 2.7, both in the “Digitalisation for healthcare services” group (Figure 2).

The mean score by country ranged from 1.9 (Hong Kong) to 4.7 (India). The variation coefficient calculated on the whole scores set attributed by each country ranged from 15.4% to 44.5%, with South Korea and Hong Kong respectively minimally and maximally differentiating the attribution of scores to the individual items examined in the survey.

### Topic 1: health technology assessment (HTA) and transformation policies for digitalisation healthcare

**Figure 3:**
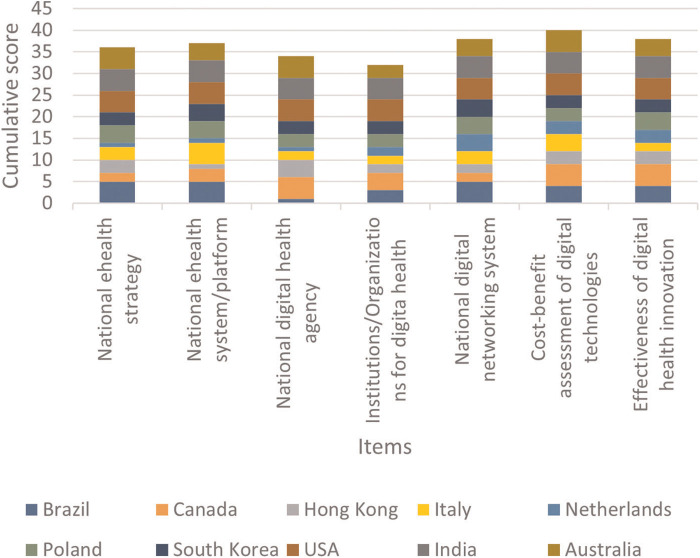
Cumulative score per proposed item of Topic 1.

In the “HTA and transformation policies for digitalisation of healthcare” section (Figure 3), the countries that showed the highest coefficient were US (5.0) and India (5.0). The country with the lowest mean score was the Netherlands (2.1).

In regard to how countries responded on the various items, the highest mean score was observed for the “cost-benefit assessment of digital technologies” (4.0) and the lowest for the “institutions/organisations for digital health” (3.2). The highest standard deviation value was observed for “national digital health agency” since Brazil and the Netherlands reported a score of one (Supplementary Table S1).

### Topic 2: development of digital health integration

**Figure 4:**
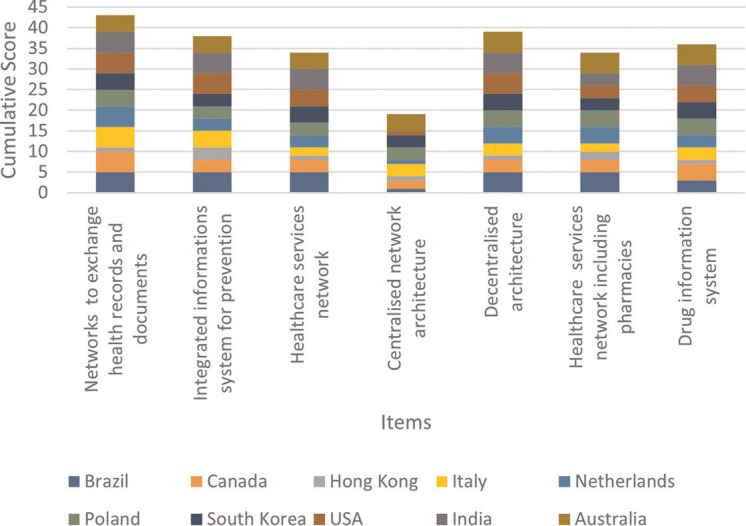
Cumulative score per proposed item of Topic 2.

In the second topic, “Development of digital health integration” (Figure 4), “*networks to exchange health records and documents*” got the highest score (4.3), while “*centralised network architecture*” received the lowest ones (2.3). The low values of standard deviations between the ten country means, ranging from 0.9 to 1.3, indicate that the ten countries attributed relatively uniform scores to the 7 items.

### Topic 3: digitalisation of healthcare services

**Figure 5:**
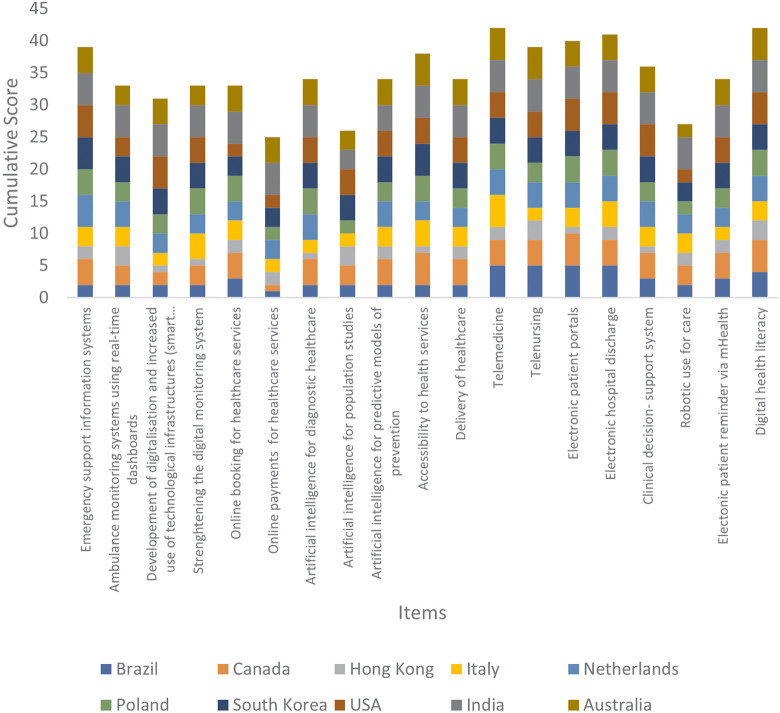
Cumulative score per proposed item of Topic 3.

Within the third topic, “*Digitalisation of healthcare services*” (Figure 5), the highest priorities were attributed to “telemedicine” and “digital health literacy” (both with a mean score of 4.2) and the lowest priority was “online payments for healthcare services” (with a mean score of 2.5).

The relatively low standard deviation of about 0.9 that was associated to the item means suggests that these assessments were generally shared by all countries.

### Multivariate analyses

**Figure 6:**
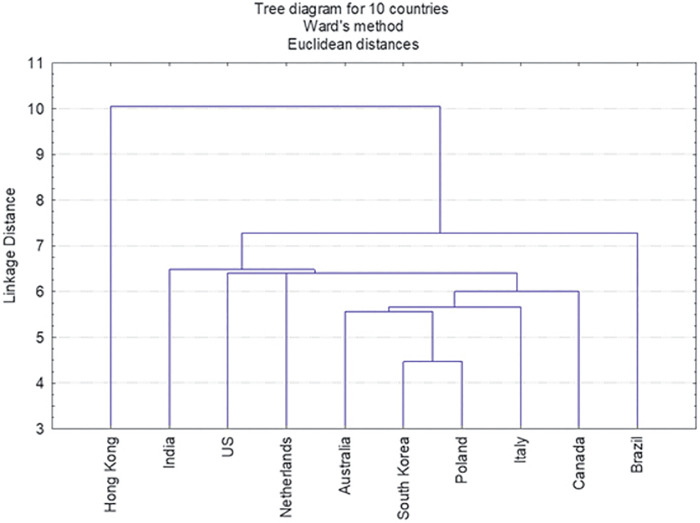
Dendrogram of the ten countries clustered by given priority identification scores.

To compare the cross-country priorities, a cluster analysis was performed, and the cluster tree (or dendrogram) (Figure 6) is based on the 330 survey outcomes (10 Countries and 33 items) (Supplementary Table S1) to classify ten world countries according to the 33 items’ scores. Hong Kong was located in a separate branch of the dendrogram because it attributed the lowest mean values to the sum of items on average (see Tables 1–3). Two other clusters were observed, the first one including India and the US which each attributed high scores to Topics 1 and 2. A second major cluster consisted of all other countries (the Netherlands, Australia, South Korea, Poland, Canada, Italy and Brazil) which attributed average and similar scores to all topics.

**Table 2:** Pairwise square Mahalanobis distances (plain text) between national priorities and corresponding probability values (italics).

	Topic 1	Topic 2	Topic 3
Topic 1		11.14032	12.53191
Topic 2	*0* *.* *0252*		4.37224
Topic 3	*0* *.* *0018*	*0* *.* *1854*	

**Table 3:** Standardized coefficients of canonical variables.

	**Root 1**	**Root 2**
Brazil	0.035	0.510
Canada	−0.150	−0.093
Hong Kong	0.200	**−0** **.** **899**
Italy	−0.019	0.105
Netherlands	−0.782	0.146
Poland	0.589	−0.278
South Korea	−0.851	−0.620
US	**0** **.** **962**	−0.126
India	0.200	−0.448
Australia	−0.064	0.602
Eigenvalues	2.142	0.680
Cumulative explained variance (%)	75.9	100.0

**Figure 7:**
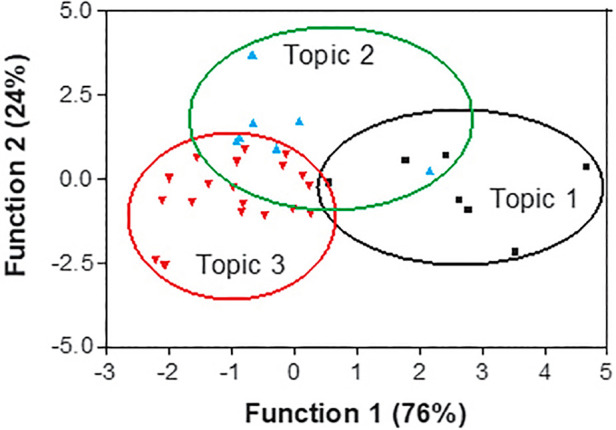
Canonical discriminant analysis plot for the three topics.

Bold values highlight countries scores which mainly affect the horizontal and vertical separation of clusters in figure 7.

#### Discriminant analysis

To compare the cross-country priorities, a discriminant analysis was performed to ascertain which pool of countries differentially rated the three topics. The significant *F* values (*F* = 2.944865; *p* < 0.0016) and the small values of Wilks’ lambda (0.17327) was calculated for the discriminating function and significatively differentiated the three topics.

The pairwise square Mahalanobis distances (Table 2) with the relative probabilities and the scatter plot diagram of the canonical coefficients (Figure 7) also show that the items included in the Topics 2 and 3 had similar scores, while there were significant differences in the priority some countries gave to Topic 3 (Digitalisation of healthcare services) and Topic 1 [Health Technology Assessment (HTA) and transformation policies for digitalisation healthcare].

Based on the absolute values of standardised b coefficients shown in Table 3, the countries that mainly affected the horizontal separation of clusters shown in Figure 7 and have different scores for Topics 1 and 3 are the US, Hong Kong, and the Netherlands. This is because the US has the highest priority for Topic 1 (mean score = 5) and Topic 2 (mean score = 3.95), while South Korea and the Netherlands gave lower scores to the same topics. Hong Kong mainly affected the vertical separation of cluster shown in Figure 7 because it gives very low scores to every item, especially items included in Topic 2.

## Discussion

Digitalisation of healthcare is universally recognized as a hot political topic, especially after the challenges that the pandemic emergency brought countries to face in terms of population needs and the quality of care (20). The world is now addressing the need to heal a difficult socioeconomical situation and is making difficult decisions during a period of great change. The European Union “Next Generation EU” Program and the US’ “Coronavirus State and Local Fiscal Recovery Funds (SLFRF)” Program are two examples of how central banks are investing large amounts of money to support a swift recovery. In the EU, a recovery and resilience plan has been produced by all of the countries benefiting from the upcoming funds. Even though the official plans could still undergo changes, different countries such as Austria, Belgium, Croatia, Czech Republic, Finland, France, Greece, Hungary, Italy, Romany, Slovakia, Slovenia and Spain have all planned to invest in digitalising their healthcare (21). In some cases, National agencies are planned to address the difficulties that the digitisation of healthcare might entail (22–24).

The priorities identification section is the starting point for addressing the answers with a rational scheme. It aims to highlight critical points collected by single countries in the process of digitalisation of health.

Previous GDHP literature already described GDHP members’ state of affairs on digital health by utilizing surveys, showing their utility in monitoring and detecting the efficacy and adaptability of this method in 2019 (12, 14, 20, 25, 26) and 2020 (9, 27–29). Our study is novel as it provides unique insights into countries with different socio-economic statuses working together to approach the digital transition of national health systems.

Results from each section may help define whether individual countries are willing and aiming to address economical resources to the development of the suggested items, whether they share interests and objectives as well as how they are reacting to digital innovations.

The ten countries’ mean score of 3.54 on the whole data set of 33 questions, calculated with a variation coefficient of 22.4%, suggests that the attention to a digital evolution in health is shared by most of the evaluated countries.

This study highlighted that, for priority-setting in digital health, most of national institutions pointed to similar objectives and interests. Some of the involved countries, especially India and the US, showed high interest on the topic by assigning high scores to most of the discussed items. Alternatively, Hong Kong had a total mean score of 1.9, indicating the digitalisation of health might not be considered a priority by local authorities, as stated in literature as well (30). Based on survey results and literature analysis we might assume that they either already have strong digitalized healthcare services that don't need further improvements at the moment, or they have low interest in the process.

The most voted item “Networks to exchange health records and documents” demonstrates the importance that countries attribute to an electronic health record (EHR), since many scientific studies have already demonstrated how it minimizes errors, saves unnecessary time, and prevents money from being wasted on processing medical data (31). Likewise, the obtained score emphasises the importance that the involved countries attribute to interoperability and their interest in system-wide health information exchange. Many countries, including Italy (32), are already trying to fully digitalize data, confirming it to be one of the hottest current topics. The Australian Department of Health has established the Connected Health Data (CHD) Program, a Data Integration Partnership Australia initiative with the aim to build a safe and secure platform for managing data access through Health's existing Enterprise Data Warehouse (33).

Under the “Digitalisation of healthcare services” topic, both “Telemedicine” and “Digital health literacy” received a mean score of 4.2. Telemedicine is popular in both medicine and politics, with a large number of studies defining its improvements in the areas of efficiency, patient experience, and clinician experience (34–36). As a result, there is a need to prioritize this topic on experts task forces. Digital health literacy, defined by WHO as the ability to seek, find, understand, and appraise health information from electronic sources and apply the knowledge gained to addressing or solving a health problem (37), however, might be considered a more controversial item. The patients ability to face with technical medicine topics could in fact generate misunderstandings and contrasts, slowing the process of care (38). Even though different efforts have been made to help the patient approach a disease in a more conscious way worldwide (39), its safety has not been adequately addressed, especially when referring to the need of a constant upgrade and an adequate communication method. A longer time might be needed before this skill could be mastered by a wider audience (40).

Most of the differences among evaluated countries regard Topic 1, specifically concerning the item “National digital health agency.” This section of the survey explores countries attitude towards Health Technology Assessment (HTA) and transformation policies for digitalisation healthcare. The poll refers to e-health as to an umbrella term including the use of information and communication technologies in the healthcare space. Inquiries citing digital health, instead, intended to examine their specific approach to technologies intersecting with health. The different results could reflect the different political assets of the respondents and requires further thoughts and meetings about the item. For example, Australia and India attributed the maximum score to the development of a national digital health agency while Brazil and the Netherlands attributed the lowest. This response reflects the programs put in place in those countries: Australia established the Australian Digital Health Agency, a corporate Commonwealth entity with the mission to create a collaborative environment to accelerate adoption and use of innovative digital services and technologies (22); the Indian National Health Authority instituted the Ayushman Bharat Digital Mission (ABDM), with the aim of developing the backbone necessary to support the integrated digital health infrastructure of the country (41). A list of national e-health strategies adopted by the included countries can be found in supplementary material (see Supplementary Table S2).

Surprisingly, more than one country assigned a lower-than-average score to the item “artificial intelligence (AI) for predictive models”. Predictive models, fundament of personalised medicine, can be useful tools both for prevention and during the phase of treatment of a disease, whose utility might not have been evaluated by these countries yet. The ability to model the management of a patient on its specifics could maximize the effectiveness of treatment (42). This could be an interesting subject to discuss in a future GDHP summit. AI can be considered an important ally in prevention and different studies have defined it as easy to implement. Unlike the diagnostic support field, where the application of AI still leaves room for doubts about its safety, predictive models have already shown the ability to improve the efficiency, security and quality of preventive healthcare interventions (43) leading to the need to invest and prioritize this field.

On the same row, with a mean score of only 2.6, the “Artificial Intelligence for population studies” item strengthens the conclusion that most of the surveyed countries might not have evaluated the cost-benefit ratio of the application of digital health to the prevention field, although multiple studies have demonstrated how even simple tools like social networks could support prevention campaigns (such as vaccination campaigns) to reduce population risks due to pandemics (44).

Health monitoring is a fundamental aspect of preventive medicine, helping with the early detection of diseases and reducing suffering and medical costs. The diagnosis and prompt treatment of diseases can drastically influence the evolution of a patient's clinical history (43, 45).

A similar result can be seen in the in the “Drug information system” and “Strengthening the digital monitoring system” sections, with a mean score of 3.6 and 3.3, respectively. The definition of a system supported by AI for the administration of drugs might result in easier and safer access to medications but also in a more competent interoperability between countries in the fight against illicit usage of medications. The U.S. Food and Drug Administration (FDA) has already promoted the international harmonization of approaches for expediting the global adoption of emerging technologies in the drug monitoring system in order to avoid drug shortages (46). Different examples of AI applications in the illicit traffic and usage of substances can be found in literature (47).

### Strengths and limitations

The main strength of this study is its unique value. There is currently no similar international forum to share best practices and enable co-working in digital health. Sharing this survey with politic representatives ensures a more rigorous construction of the answers. The publication of the whitepaper on the development of standard benefits and outcome measurements (11) guided the researchers in following a coherent path to the previous GDHP literature and hope similarly guide others with our work here. Finally, the heterogeneous sample derived from distinct geographical regions gives a worldwide representation of the topics.

There are limitations to our study. As with any voluntary surveys, participation bias, or non-response bias is a limitation. Those with strong opinions may have been more likely to respond to the survey than those that did not feel strongly about the subject matter and may skew responses. In this study, there was a low response rate of 34.5% (*n* = 10). Dividing the reached countries with a geographical method, the response of African countries was the lowest (0 out of 2 countries). The response of North America was the highest (2 out of 2 countries), followed by Oceania (1 out of 2 countries), Asia (3 out of 9 countries), Europe (3 out of 10 countries) and South America (1 out of 4 countries). However, the different representation of the areas in the partnership doesn't allow us to statistically evaluate these numbers. This section of the survey included 33 questions and required a pragmatic study of national needs to be answered. The length of the survey may have discouraged responses therefore limiting sample size to those with adequate time. This survey also lacked a scientifically validated method during its development due to the novelty of this topic, therefore more studies are needed to confirm the current data. Finally, the low responses number did not allow a generalisation of results.

## Conclusion

The resulting insights on the differences between digital health priorities identification among different GDHP countries serves as a starting point to coordinate further progress on digital health worldwide and will foster evidence-based collaboration. The collection of evidence in this field, arising from the implementation of the identified priority actions, will be a fundamental tool for a fair development of individual countries as much as their interoperability is. Similarly, the application of HTA in the field of digital technologies should be considered an activity to be promoted in the future for its fundamental contribution not only in retrospectively defining evidence, but also in informing and supporting decisions within health services to improve the quality of care. Since this survey highlighted different shared priorities, further discussions might focus on identifying common, well-defined, and standardized objectives. This would allow the development and application of shareable and interoperable digital health services at an international level. With greater spread and advances of digital health technologies, we believe that a positive impact will be seen in the fight against inequalities and in the quality of care worldwide by breaking down the barriers of availability, accessibility, and affordability.

## Data Availability

The original contributions presented in the study are included in the article/**Supplementary Material**, further inquiries can be directed to the corresponding author/s.
