# Supplementary material for: Approaches to priority identification in digital health in ten countries of the Global Digital Health Partnership
Source: Front Digit Health. 2022 Sep 16;4:968953. doi: 10.3389/fdgth.2022.968953 (PMC9632991; doi:10.3389/fdgth.2022.968953)
Supplement: Supplementary file 1 [file Datasheet1.docx]

**Search string**

(“digital health” OR “health digitalisation” OR “WHO framework” OR “digital competence framework”) AND (“national plan” OR “national strategy” OR “national intervention”) AND (“questionnaire” OR “survey” OR “pool”) AND “acceptability”
