# Supplementary material for: Approaches to priority identification in digital health in ten countries of the Global Digital Health Partnership
Source: Front Digit Health. 2022 Sep 16;4:968953. doi: 10.3389/fdgth.2022.968953 (PMC9632991; doi:10.3389/fdgth.2022.968953)
Supplement: Supplementary file 2 [file Table1.docx]

**Supplementary Material**

**Supplementary Tables**

| **Table 1- Involved stakeholders per country** | | |
| --- | --- | --- |
| **GDHP Participant Country** | **Country GDHP Reference Point** | **Organization** |
| Australia | Amy Winter  Senior Policy Advisor  Rick Sondalini  Senior Policy Advisor | Australian Digital Health Agency |
| Brazil | Dr Márcia Elizabeth Marinho da Silva  General Coordination of Innovation in Digital Systems  Thaís Lucena de Oliveira  General Coordination of Innovation in Digital Systems | Ministry of Health, Department of Informatics (DATASUS) |
| Canada | Michael Green  President and CEO  Simon Hagens  Senior Director, Performance Analytics | Canada Health Infoway |
| Hong kong | Dr Ngai Tseung Cheung  Head IT&Health Informatics  Dr Joan Hung  Principal Assistant Secretary | Electronic Health Record Office, Food & Health Bureau, Government of Hong Kong |
| Italy | Dr Fidelia Cascini  Digital Health Expert | Italian Ministry of Health  Directorate for Digitisation, Health Information System and Statistics (DGSISS) |
| India | Lav Jaiswal  Director (eHealth)  Lav Agarwal  Joint Secretary | Ministry of Health and Family Welfare, Government of India |
| Netherlands | Herko Coomans  International Digital Health Coordinator  Roger Lim  Senior Policy Officer | Ministry of Health, Welfare and Sport, The Netherlands |
| Poland | Hubert Życiński  Head of Unit | Ministry of Health  Department of Innovation |
| South Korea | Dr Hun-Sung Kim  Associate Professor | The Catholic University of Korea |
| United States of America | Dr Aisha Hasan  Head of Global Health IT | U.S. Department of Health and Human Services |

| **Table 2- National e-health strategies** | |
| --- | --- |
| Country | National e-health strategy |
| Australia | The Australian Digital Health Agency has been established by the governments of Australia  with a remit to evolve digital health capability through innovation, collaboration and leadership to facilitate digital health integration in the health system. The Agency has developed the National Digital Health Strategy through extensive consultation with the Australian community and comprehensive analysis of the evidence. Governments continue to make significant investments in digital health systems. State and territory governments are embarking on major projects to implement state and territory-wide electronic medical records and to achieve integration across the range of clinical information systems in hospitals and health services managed by state or territory governments.(1) |
| Brazil | The National Digital Health Strategy Action, Monitoring and Evaluation Plan (PAM&E) is intended to identify, prioritize, and integrate, in a coordinated way, healthcare programs, projects, and actions, information and communication services and systems, financing mechanisms, infrastructure, governance, technologies, and human resources, aimed at achieving the ESD vision, in which it plays an integrating part.(2) |
| Canada | Nationally, Canada Health Infoway (Infoway) has been created to fund, coordinate and work with the provinces to implement interoperable Electronic Health Record and Telehealth solutions across the country. Infoway is an independent, not-for-profit corporation and partnership of federal, provincial, and territorial governments. The Infoway mission is to foster and accelerate the development and adoption of eHealth information systems with compatible standards and communication technologies, on a pan-Canadian basis, with tangible benefits to Canadians.(3) |
| Hong Kong | In Hong Kong, there are several ongoing digital healthcare platforms and digital health projects, such as he Electronic Health Record Sharing System (eHRSS), launched in 2016, voluntary for both practitioners and patients; DoctorNow, a telemedicine service provider offering remote healthcare and Video consultations; Pulse by Prudential, a digital health app offering telemedicine, AI assessment, medicine delivery and connecting registered users to nearby clinics; Quality HealthCare Patient Portal, an integrated online platform for digital ticketing and appointment booking as well as video consultation for telemedicine. Although the government has stated its policy to prioritise the implementation of telehealth services in public hospitals, a gap remains between what healthcare services in Hong Kong could be and the situation as it stands.(4) |
| India | India took big strides towards empowering the nation with E-Health. Since 2015, a National eHealth Authority (NeHA) promotes, regulates and sets standards for eHealth. The National Telemedicine Service “eSanjeevani” was rolled out in November 2019 as an important component of the Ayushman Bharat Health and Wellness Centre (AB-HWCs) programme. It is a digital health initiative of the Ministry that supports two types of teleconsultation services-Doctor-to-Doctor (eSanjeevani) and Patient-to-Doctor (eSanjeevani OPD) Tele-consultations. In 2019, the Ministry of Health and Family Welfare released National Digital Health Blueprint (NDHB) as an architectural framework for the effective implementation of Digital Health interventions. With a vision to create a national digital health ecosystem, in 2020 the launch of the National Digital Health Mission was announced.(5) |
| Italy | In 2021, the Interministerial Committee for Digital Transition attributed to the National Agency for Regional Health Services (AGENAS) the task of managing the implementation of the digitalization process at the national level, one of the pillars of National Recovery and Resilience Plan (NRRP). The NRRP addresses the need to strengthen the National Health System through the link with hospital-based healthcare of community structures and facilities, such as with community homes and community hospitals. Goals are focused on the improvement of home care, telemedicine, and e-health services. Further improvements of the NRRP are linked to the completion and systematic use of the Personal Electronic Health Record (PEHR), and to a better delivery and monitoring capacity of the Essential Levels of Assistance (LEA) through more effective information technology tools and systems. Finally, Italian institutions aim to economically support the ongoing digital update of hospitals and technological equipment(6) |
| Netherlands | Netherlands has recognised the importance of the role of digital health (eHealth) in addressing the pressing issues facing the Dutch healthcare system, being an ageing population, rising healthcare costs, and a shortage of competent medical staff. In 2021 the Dutch Healthcare Authority (NZa) increased funding for eHealth. This important development means that the costs of telehealth – for example an online consultation by video link – can be reimbursed in the same way as face-to-face healthcare services provided by a general practitioner (GP) or a medical specialist. Current overarching themes that the Health Ministry focuses on are the transition from traditional hospital-based care to homecare, increasing patient involvement in the care process, and facilitating the safe exchange of patient information between providers, and between provider and patient.(7) |
| Poland | In Poland strategy for eHealth is connected mainly to medical information systems. Little attention is given to its clinical aspects. Its development came into being largely because of EU requirements. Actions undertaken by the state and its institutions are the main factors in eHealth development as far as legislation and standard setting are concerned. Regrettably, these institutions have so far failed to deliver. Although exact causes are hard to pinpoint, persistent miscommunications and failure to define clear areas of responsibility between the Ministry of Health and its own agency CSIOZ have largely contributed to the situation. (8) |
| South Korea | South Korea has become a global leader in Information and Communication Technologies, and as part of its preparations for the Fourth Industrial Revolution, the country is promoting national health and medical informatization in order to provide safer and higher-quality medical services anytime and anywhere through the use of big data in the medical field. Most hospitals have implemented electronic medical record (EMR), as well as personal health records (PHR). Hospitals have implemented around three to seven government-sponsored information/data transmission and receiving systems for statistical or investigative objectives. For secondary usage of medical data, more than half of tertiary hospitals have implemented a clinical data warehouse or shared data model.(9) |
| US | With the signing of the Health Information Technology for Economic and Clinical Health (HITECH) Act of 2009, President Barak Obama changed the face of healthcare. The legislation provided financial incentives for providers to implement health information technologies and financial penalties for those who did not. Development of eHealth applications is however mostly pulled by private agencies and insurances. The digitalisation process is nearly all done in the private sector, primarily by the vendor community. The role of the federal system has been seen as to promote agreement on and identification of specific standards, coordination of activities. As for telemedicine, although most hospitals already use some kinds of technology to keep track of patients, to be eligible for Medicare reimbursement, a case must conform to a set of national guidelines; namely, the receiver of telemedicine service must be in a rural geographical location defined as either a Health Professional Shortage Area or a county outside of a Metropolitan Statistical Area.(3) |

Bibliography

1. Australia’s National Digital Health [ADHA]. Digital health strategy. 2020;(July):3–81. Available from: https://ehealthresearch.no/files/documents/Undersider/WHO-Symposium-2019/1-3-Skovgaard-ENG.pdf

2. Brazil’ s National Digital Health Strategy Action , Monitoring and Evaluation Plan. 2023.

3. Borycki EM, Newsham D, Bates DW. eHealth in North America. Yearb Med Inform. 2013;8:103–6.

4. Digital Health for Hong Kong and the Greater Bay Area: A briefing paper for the Chief Executive of the Hong Kong SAR Government by the Healthcare Committee of the British Chamber of Commerce in Hong Kong.

5. Strengthening the Roots of India’s Health Sector; Major Initiatives by Ministry of Health, PNBS

6. Filippini T, Vinceti SR. Italian National Recovery and Resilience Plan: a Healthcare Renaissance after the COVID-19 crisis? Acta Biomed. 2021;92(i).

7. THE DUTCH E-HEALTH SECTOR, Commissioned by the Netherlands Enterprise Agency, Ministry of National Affairs.

8. Kautsch M, Lichoń M, Matuszak N. Development of publicly funded ehealth in Poland: Barriers and opportunities. Econ Sociol. 2016;9(3):28–40.

9. Lee K, Seo L, Yoon D, Yang K, Yi J-E, Kim Y, et al. Digital Health Profile of South Korea: A Cross Sectional Study. Int J Environ Res Public Health. 2022;19(10):6329.

**Supplementary figures**

Figure 1 - Evaluated items, scores and means

**Search string**

(“digital health” OR “health digitalisation” OR “WHO framework” OR “digital competence framework”) AND (“national plan” OR “national strategy” OR “national intervention”) AND (“questionnaire” OR “survey” OR “pool”) AND “acceptability”
